# γδ TCR Recognition of MR1: Adapting to Life on the Flip Side

**DOI:** 10.1016/j.tibs.2020.03.012

**Published:** 2020-07

**Authors:** Benjamin E. Willcox, Fiyaz Mohammed, Carrie R. Willcox

**Affiliations:** 1 Institute of Immunology and Immunotherapy, University of Birmingham, Birmingham, UK; 2 Cancer Immunology and Immunotherapy Centre, University of Birmingham, Birmingham, UK

**Keywords:** T cell receptor (TCR), gamma delta, major histocompatibility complex (MHC), complementarity determining region (CDR)

## Abstract

Nonclassical class I MHC-like molecules are ligands for several unconventional T cell populations. Recently, Le Nours *et al*. identified human γδ T cells recognising MHC-related protein-1 (MR1) via their T cell receptor (TCR). Also recognised by the αβ-TCR of mucosal associated invariant T cells, MR1 interacts with specific γδ-TCRs using strikingly diverse binding modes, suggesting fundamental differences in γδ T cell recognition.

γδ T cells, defined by their surface expression of paired γ and δ T cell receptor (TCR) chain heterodimers, have been retained throughout vertebrate evolution and play critical roles in host immunity in diverse settings, including infection, antitumour immunity, and immune regulation [1,2]. They are also of increasing therapeutic interest. Although it is widely accepted that unlike αβ T cells, they do not recognise peptide-MHC molecules, the question of what antigens they do recognise via their TCR still remains substantially unresolved.

Since the discovery of γδ T cells, diverse molecules have been proposed as candidate γδ-TCR ligands [3]. While recent evidence has confirmed butyrophilin/butyrophilin-like (BTN/BTNL) family molecules as direct TCR ligands for γδ T cell populations bearing specific TCRγ chain variable regions (either Vγ4/Vγ7 [4] or Vγ9 chains [5,6]), the mouse nonclassical class I MHC molecules T10 and T22 were the first γδ-TCR ligands to be confirmed biochemically [7]. Since then, γδ T cells capable of interacting via their TCR with the nonclassical class I MHC molecule CD1d have also been defined [8].

Recently, Le Nours and colleagues have made an important step forward by demonstrating a third category of nonclassical class I MHC molecule, MHC-related protein 1 (MR1), is also a target for γδ-TCR binding [9]. Their study combines use of MR1-tetramer staining to identify MR1-binding γδ T cell populations, surface plasmon resonance (SPR) to assess direct γδ-TCR/MR1 binding, and structural techniques to establish relevant binding modes. Their findings significantly advance our understanding of MR1 and may hold some fundamental lessons regarding γδ-TCR recognition itself.

## Adaptive γδ T Cell Recognition of a Monomorphic Ligand

CD1d and MR1 are established recognition targets for defined αβ T cell populations, namely invariant natural killer T cells (iNKTs) and mucosa associated invariant T cells (MAITs), respectively. Aligning with the monomorphic nature of these ligands, both iNKTs and MAITs express a highly restricted TCR repertoire and also exhibit distinct innate-like phenotypes relative to the bulk αβ T cell compartment. Using MR1 tetramers, Le Nours *et al.* showed the situation is very different for MR1-binding γδ T cells.

In most people, MR1-specific γδ T cells comprised a low percentage (~0.1%) of γδ T cells. Strikingly, their TCR repertoire was diverse, reflecting the TCR-diverse adaptive-like Vδ2^neg^ repertoire as a whole and chiefly focussed on the prevalent Vδ1 and Vδ3 subsets, combined with a broad range of Vγ chains. Also, MR1-specific γδ T cells phenotypically resembled the entire γδ T cell pool. By contrast, the semi-invariant, innate-like Vγ9Vδ2 T cell subset, which bears a highly restricted TCR repertoire, was not a source of MR1-specific γδ T cells. These features closely mirror those of CD1d-specific γδ T cells (and γδ T cells specific for the exogenous model antigen phycoerythrin), but contrast with properties of iNKTs and MAITs. Relative to αβ T cells, γδ T cell recognition of nonclassical class I MHC molecules may therefore be fundamentally skewed towards highly TCR-diverse, adaptive-like γδ subsets, which are thought to bind a diverse array of ligands.

Whilst limited phenotypic analysis of MR1-specific γδ T cells was carried out, their differentiation status was not defined. Addressing this question, highly relevant for adaptive compartments, would clarify if MR1-specific γδ T cells reside within the T_effector_ subpopulation, consistent with *bona fide* MR1-directed adaptive T_effector_ responses, or alternatively within the T_naive_ subpopulation, which lacks effector capability and would be more suggestive of potential adaptive reactivities [10] yet to encounter MR1 *in vivo*. *In vitro* assays involving transduction of MR1-binding TCRs into Jurkat T cells showed that although CD69 upregulation was not always observed, MAP kinase/ERK kinase activation was universal, confirming a potential to support TCR triggering. Although low levels of MR1-specific T cells were detected in most individuals, MR1-tetramer-positive cells were enriched in some individual samples, including in newly diagnosed coeliac disease and Merkel cell carcinoma. This finding suggests both TCR-diverse T_naive_ and clonally focussed T_effector_ subpopulations may contribute to the MR1-specific γδ T cell pool; the latter could contribute to physiological adaptive γδ T_effector_ responses in some individuals. Future studies will no doubt shed light on these questions.

## Diverse Modes of Antigen-Agnostic γδ TCR Binding to MR1

**Figure 1:**
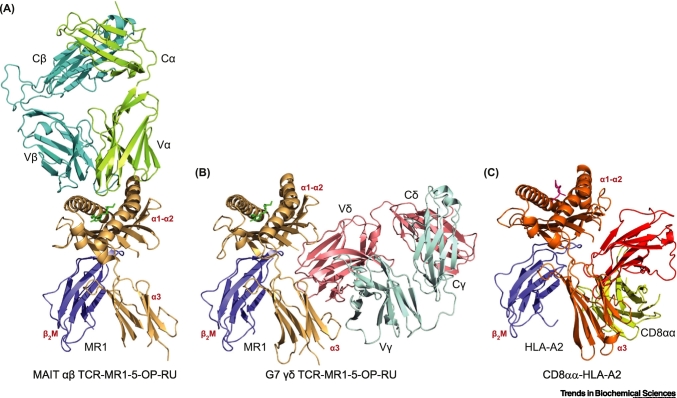
Overview of the MAIT αβ TCR-MR1-5-OP-RU, G7 γδ TCR-MR1-5-OP-RU, and CD8αα-HLA-A2 Complexes. (A) Cartoon representation of the MAIT αβ TCR-MR1-5-OP-RU complex (PDB ID: 4NQC): MR1, brown; β2-microglobulin (β_2_M), blue; 5-OP-RU, green; α-chain, light green; β-chain, cyan. (B) Cartoon representation of the G7 γδ TCR-MR1-5-OP-RU complex (PDB ID: 6MWR): MR1, brown; β_2_M, blue; 5-OP-RU, green; Vγ9 chain, pale cyan; Vδ1 chain, salmon. (C) Cartoon representation of the CD8αα-HLA-A2 complex (PDB ID: 1AKJ): HLA-A2, orange; β_2_M, blue; peptide, hot pink; CD8αα, red and yellow. Ig-like variable and constant domains for α, β, γ, and δ chains are indicated by Vα, Cα, Vβ, Cβ, Vγ, Cγ, and Vδ, Cδ respectively. Abbreviations: MAIT, mucosa associated invariant T cell; MR1, MHC-related protein 1; TCR, T cell receptor.

Le Nours and colleagues also outlined the molecular basis of γδ-TCR/MR1 interaction. SPR binding studies revealed MR1-binding γδ-TCRs tested were largely ‘antigen agnostic’ and either entirely unaffected or only slightly impacted by the presence/absence of MR1-bound antigen, suggesting potential ‘inherent autoreactivity’ to MR1-expressing cells even in the absence of antigenic challenge. Although iNKT and MAIT TCR/ligand recognition has also been linked to ‘inherent autoreactivity’, this operates via binding modes apparently exclusively involving interaction of αβ-TCR CDR loops with the α1α2 platform (Figure 1A). Moreover, conserved iNKT and MAIT TCR V-region usage and respective germline-encoded CDR1/2 loops provide a clear basis for such semi-invariant interactions, which appear literally and immunologically ‘restricted’ to the α1α2 platform of CD1d or MR1, allowing potential for discriminating presence/absence and nature of bound antigen.

By contrast, mutational analyses suggested that collectively, the MR1-binding γδ-TCR pool was not limited to interaction with the upper-face of the α1α2 platform, but also contained TCR specificities recognising the membrane-proximal ‘flip-side’ of MR1, predominantly to the α3 domain. X-ray crystallographic analysis confirmed this highly novel binding mode. Importantly, ‘flip-side’ interaction was consistent with antigen ‘agnosticism’ and involved no contacts to upper-facing α1α2 helical platform residues, instead predominantly featuring α3 domain contacts, with additional interactions to the platform’s underside (Figure 1B). Consistent with diverse Vγ usage in the MR1-binding γδ-TCR pool, interaction was dominated by Vδ-mediated contacts. Moreover, while some CDR1δ-mediated involvement was evident, Vδ interactions involved critical hydrophobic contacts formed by CDR3δ residues, consistent with only a small proportion of the extremely diverse Vδ1 TCR repertoire satisfying the molecular criteria for MR1 recognition. This mode resembled CD8αα/class I MHC recognition (Figure 1C), which itself was likened to antibody/antigen interaction [11]. These observations confirm that γδ T cell recognition of MR1 is indeed fundamentally different to CD1d/MR1-restricted recognition by semi-invariant iNKTs and MAITs.

In summary, the identification of MR1-binding γδ T cells is a significant advance for both MR1 and γδ T cell biology and should be applauded. By contrast to iNKTs and MAITs that now have established contributions to immune regulation, including in diverse models of infection/disease, the physiological role and importance of γδ T cells that recognise nonclassical class I MHC molecules has remained largely unclear since their initial identification 20 years ago. In this context, the immunobiological meaning and relevance of antigen-agnostic recognition of MR1 by γδ T cells is currently unclear. Moreover, future studies should consider the parallel and nonmutually exclusive possibilities that MR1 interactions with γδ-TCRs either contribute to physiological adaptive γδ T cell effector immune responses, or alternatively in some cases largely represent potential autoreactivities. In this second scenario, the presence of MR1-specific cells may reflect the fundamental potential of the adaptive γδ-TCR repertoire to recognise diverse self-antigens, from which particular autoreactive TCR specificities may be selected to differentiate into T_effector_ cells to support adaptive γδ T cell immunosurveillance following relevant immune challenges. Given the recent finding that MR1-directed αβ-TCR alloreactive recognition of an antigenically altered form of MR1 can mediate broad antitumour responses [12], it is tempting to speculate on the potential relevance of γδ-TCR/MR1 interactions in such settings, particularly given established *in vitro* antitumour capabilities of γδ T cells. The study by Le Nours and colleagues is a fundamental step forward that should pave the way for future studies to address such fascinating questions.
